# Sexual orientation knowledge and attitudes and its association with therapy satisfaction among lesbian, gay, and bisexual + Hispanic Puerto Ricans

**DOI:** 10.1186/s12889-023-15811-8

**Published:** 2023-05-11

**Authors:** Caleb Esteban, Margarita Francia-Martínez, Miguel Vázquez-Rivera, Frances Crespo, Taysha Bruno-Ortiz, Aquiria M. Santiago-Ortiz, Alfonso Martínez-Taboas

**Affiliations:** 1 Clinical Psychology Program, School of Behavioral and Brain Sciences, Ponce Health Sciences University, PO BOX 7004, 00732-7004 Ponce, Puerto Rico; 2 Carlos Albizu University, San Juan, Puerto Rico; 3 Ana G. Méndez University System, San Juan, Puerto Rico; 4 Instituto de Investigación y Desarrollo para Estudiantes Dotados, San Juan, Puerto Rico; 5 University of Puerto Rico, Río Piedras Campus, Puerto Rico; 6 InterAmerican University of Puerto Rico, San Juan, Puerto Rico

**Keywords:** LGB/LGBT Health, Therapy, Knowledge, Attitudes, Mental health, Sexual minorities health, Stigma

## Abstract

This study aimed to examine the difference in therapy satisfaction between lesbian, gay, and bisexual + (LGB +) individuals and heterosexual individuals, and to identify the association between therapy satisfaction and the perception of knowledge and attitudes of their last therapist among the LGB + participants. Through an exploratory design with a comparative group, 125 LGB + and 75 heterosexual participants were recruited online by availability. Results indicate that the participants’ sexual orientation has no significant relation on therapy satisfaction. However, there was a significant positive association between satisfaction with therapy and the LGB + participants’ perception that their therapist demonstrated knowledge and positive attitudes. This research highlights the importance for continuous education and curriculum efforts on LGB + issues.

Stigma, exclusion, and discrimination toward non-heterosexual persons has been maintained by social, religious, and legal institutions throughout many cultures around the world [1, 2]. Stigma and its related sequelae are observed in many instances of everyday life and social institutions, such as cultural scripts, legal systems, public and private institutions, and in different religious groups [3]. These become more prominent as negative attitudes and prejudice toward lesbian, gay, and bisexual umbrella identities [e.g., bisexual, pansexual] (LGB +) are legitimized [4]. Mental health professionals are not exempt from promoting discrimination. The American Psychiatric Association [APA] maintained that homosexuality was a psychiatric diagnosis until 1973, when the diagnosis was excluded from the Diagnostic and Statistical Manual of Mental Disorders (DSM) [5]. Despite the political movements for the equal rights of sexual minorities, it is still a challenge to avoid the damage caused by the stigma that some therapists perpetuate when providing services to Hispanic LGB + individuals.

There is considerable agreement, among therapy researchers and practitioners, that the therapist-client/patient relationship plays a crucial role in the therapeutic process and outcome [6]. On the other hand, it is not uncommon for LGB + clients/patients to experience unhelpful therapy practices such as heterosexism, lack of knowledge about issues unique to being a sexual minority, and the dismissal of sexual orientation on psychological functioning [3, 7]. To promote more competent, sensible, and adequate therapeutic services for LGB + individuals, it is important to expand the knowledge on affirmative model services. The affirmative model is a concept in which therapists are aware of their own attitudes and beliefs and understand the impact of their own biases and the dynamics of discrimination, stereotypes, power, privilege, and oppression when working with LGBTQ + individuals [3].

## Research in Puerto Rico

In Puerto Rico, as a Hispanic culture, several studies have found that certain therapists exhibit stigma toward LGB + individuals, affecting bisexual individuals the most [8, 9]. These findings about the general concern of bias toward LGB + individuals have provided useful guidance for a more informed psychology practice such as the creation of the Standards to Work and Intervene with the Lesbian, Gay, Bisexual and Trans Identities Community of the Puerto Rico Psychological Association [PRPA] [10, 11].

Although there is ample literature on LGB + issues in other parts of the world, this is still an underdeveloped field in Puerto Rico and other Hispanic countries. In a recent literature review, Martínez-Taboas [11] stated that the first professional publication in Puerto Rico on LGBT + issues was published in 2003. Since then, several journal articles and dissertations have been added to the field. In a recent publication, Esteban [12] reviewed investigations related to gay men’s issues in Puerto Rico from 2000 to 2019. They found that 41% of the publications were related to stigma, prejudice, and social distance toward gay men. Other themes were sexual identity development, religion/spirituality violence, and alcohol and substance use. Notably, there is an absence of studies on positive factors and aspects related to the best practice and services with LGB + clients/patients, which can help eliminate health disparities.

In studies regarding the attitudes of mental health professionals, specifically in Puerto Rico, it has been found that around 16% of psychology graduate students show anxiety during therapeutic encounters with gay and lesbian persons, 13% choose to refer lesbian and gay clients/patients cases before offering them therapeutic services, and 16% negatively self-evaluate their clinical skills with gay and lesbian clients/patients [13, 14]. These numbers warn us of a group of therapists in Puerto Rico that may lack skills, competence, and knowledge to work with LGB + clients/patients; therefore, increasing the risk of harm, revictimization, and contribute to mental health disparities.

### Health disparities among LGB + 

Abundant research suggests that LGB + individuals suffer from psychological symptoms such as depression, anxiety, suicide, sexual, physical, and verbal abuse, and problematic use of alcohol, drugs, and tobacco [4, 15–17]. Some of these symptoms have been related to minority stress [18]. Meyer proposed the minority stress theory, which mainly advances the idea that health disparities among minorities can be largely explained t by stressors induced by a hostile, harassing, and discriminating culture and environment [11]. This model has been widely used to explain the higher rates of mental health issues and disparities shown by LGB + individuals [19].

Meyer [20] proposed that the minority stress concept can be described along a distal to proximal continuum. Distal stressors refer to external events that impacted the individual (e.g., negative life events, microaggressions, social stigma, discrimination, and victimization). Proximal stressors are related to the personal appraisal of the LGB + individual, specifically the fear of revealing their identity, the inability to confront different types of rejections, and the internalization of LGB + phobias.

In addition, a study conducted by Vázquez-Rivera [13], found that, in their sample of 220 Hispanic graduate psychology students, 70% reported not receiving any formal education nor graduate courses on topics related to sexual minority issues. Similar findings were reported by Esteban years later [21]. Lack of knowledge and competencies regarding sexual minority issues has been associated with increased risks of unhelpful therapeutic practices and negative perceptions of the therapeutic process by LGB + clients/patients [3].

### Therapeutic experiences of LGB + Clients/Patients

Therapy should not become a source of oppression for disadvantaged populations such as LGB + individuals. A person receiving therapy from a professional that perpetuates societal myths and stigma can develop iatrogenic harm and worsen their symptoms. Some investigations support the view that most LGB + individuals evaluate therapists during their first appointment in order to verify that they show affirmative attitudes about their sexual orientation and will be responsive to their needs [2].

Several studies have confirmed the negative experiences of LGB + people in therapy. McCann and Sharek [2] found that, of the sample that disclosed their sexual orientation to their therapists, 64% felt that their therapists lacked knowledge in LGB + issues, and 43% felt that the professionals did not meet their needs. Another 17% said that they would not disclose their LGB + identity to mental health professionals for fear of a negative reaction. Other studies have found that 25% of their samples perceived a lack of knowledge regarding LGB + issues from their therapists, and 21% reported that their therapists ignored their sexual orientation and/or viewed it as problematic [22].

On the other hand, studies have found that some basic therapeutic skills, the therapeutic relationship, the professional background, and the therapist’s attitudes towards their client/patient’ sexual orientation influenced whether the service was a positive or negative experience [23]. Farmer [24] and Graham [25] found that the more competent therapists with the LGB + population (using self-reported competence measures) were those that had more exposure to LGB + clients/patients in their training and clinical practices, and those who had greater attendance to professional and continued education activities on LGB + matters.

It is necessary to evaluate the perception of satisfaction that LGB + individuals have about their therapeutic services, especially due to psychology’s own historical role in considering homosexuality and bisexuality as pathologies. Although there is a strong trend of professionals and therapists who have adopted affirmative models for working with the LGB + community, this minority’s experience regarding mental health services in Puerto Rico’s particular cultural context should be researched.

### Aims

This project draws upon the minority stress model. This model explains how socially minoritized groups, such as sexual minorities, are more likely to experience constant stress due to experiences of bias, discrimination, and marginalization. The model highlights the importance of addressing the stigma and barriers for essential services that could originate health disparities [26]. The study’s specific aims were: (1) to examine if there is a significant difference in therapy satisfaction levels between LGB + and heterosexual individuals, and (2) to identify if there is an association between therapy satisfaction and the perception of knowledge and positive attitudes of their last therapist among the LGB + participants. We hypothesized that there would be a significant difference in therapy satisfaction levels between the samples, and that there would be an association between therapy satisfaction and the perception of knowledge and positive attitudes of the LGB + participants’ last therapist.

## Method

### Design and procedure

The study had a quantitative method with a transversal exploratory design. This study was approved by the IRB of Albizu University (Sum16-05). Participants were recruited online by availability, using a flyer primarily promoted on Facebook Ads (targeting LGBT + interests such as pride, rainbow flag, LGBT news, and others). The flyer was also mailed to different LGBT + community organizations on the island for promotion. The SurveyMonkey platform was used to collect the data, including the informed consent and to complete the study’s instruments. A total of 125 LGB + individuals finalized the study. Participants who did not complete the study were removed from the database. Participants had to meet the following inclusion criteria: (1) self-identify as gay, lesbian, or bisexual + (e.g., bisexual, pansexual); (2) be 21 or over; (3) be a resident of Puerto Rico; and (4) have received therapy services at any time. All inclusion criteria were verified and confirmed with a sociodemographic questionnaire.

To answer Aim 1, a comparative group of heterosexual individuals was recruited. This group was recruited via Facebook Ads with a different, untargeted flyer. The SurveyMonkey platform was likewise used for the informed consent and to complete the study. Overall, 75 participants completed the instruments. Participants for this group had to meet similar inclusion criteria: (1) self-identify as heterosexual, (2) be 21 or over, (3) be a resident of Puerto Rico, and (4) have received therapy services.

### Instruments

Four Spanish language instruments were used to obtain the data of the LGB + sample. The instruments were: 1) Sociodemographic Questionnaire, 2) MHSIP Consumer Survey—*Spanish version*, 3) Therapy Experience Questionnaire, and 4) the Perceived Attitudes in Therapy toward Lesbian, Gay, and Bisexual Scale. The comparative group (heterosexual individuals) only completed the first two instruments (1 and 2), as the latter two (3 and 4) only catered to LGB + individuals.

### Sociodemographic questionnaire

This questionnaire was created by the researchers, and collected information such as sex, gender, sexual orientation, marital status, age, income, education, religious/spiritual affiliation, and clinical information.

### MHSIP adult consumer survey – Spanish version (MHSIP-CS)

This instrument was originally created in English and validated by the Mental Health Statistical Improvement Program (MHSIP) in 2006 [27]. The 35-item instrument addressed adult and senior populations and measured satisfaction with the therapeutic process. The instrument included access to services, quality and appropriateness of the services, effectiveness, participation in treatment planning, social connection, and functioning. In 2014, the survey was translated to Spanish and adapted and validated to the Puerto Rican population by the PITIRRE Community Initiative Program. Answers stand on a 5-point Likert scale (1 = totally agree to 5 = totally disagree). Its various subscales have the following Cronbach alphas: General Satisfaction (α = 0.87), Quality of Appropriateness (α = 0.87), Participation in the Treatment Targets (α = 0.56), Perceived Outcomes (α = 0.91), Services Access (α = 0.82), Functionality (α = 0.87), Social Connectivity (α = 0.79), and Stigma (α = 0.86). For the purpose of this research, the items were adapted to an individual therapy service and the subscales of Functionality and Social Connection were eliminated, resulting in 11 items. Some examples of the items are: I liked the mental health services I received, I would recommend my therapist to friends or family members, I felt comfortable asking questions about my treatment. Total scores ranged from 11 to 55. Lower scores indicated greater service satisfaction.

### Therapy experience questionnaire

This descriptive instrument, created by the researchers, consisted of 10 Spanish-language questions regarding the therapy experience. The questionnaire examined the following aspects of therapy and the therapist: sex, approximate age, office location (town), therapist’s profession, modality of therapy, disclosure of the therapist’s own sexual orientation and religious/spirituality affiliation, number of negative and positive experiences in therapy, disclosure of sexual orientation to the therapist, having worked on issues of sexual orientation in therapy, and having been referred to another therapist because of the participant’s sexual orientation.

### Perceived attitudes in therapy toward lesbian, gay, and bisexual scale

This scale was constructed in Spanish and validated for this study [28]. This instrument consists of 36 items and two subscales: 1) the perceived LGB + knowledge of their therapist and 2) the perceived attitudes of their therapist toward LGB + individuals. For content validity, 12 evaluators with expertise in LGBT + studies assessed the items. Only two items were eliminated for being under the Lawshe’s Content Validity Radio (CVR) adequacy. Answers stand on a 5-point Likert scale (1 = totally agree to 5 = totally disagree). Total scores ranged from 34 to 170. Lower scores indicated greater positive attitudes perceived by the therapist. This scale presented acceptable coefficients with a Cronbach alfa of 0.84.

### Data analysis

A total of 107 subjects were required to achieve a statistical power of 95% to determine a correlation between predictors (knowledge and attitudes) and outcomes (therapy satisfaction), assuming an effect size *f*^*2*^ of 0.15 (medium effect size) and an alpha of 0.05 using a Bonferroni adjustment and assuming a correlation between predictors of 0.50. Each sexual orientation strata included a minimum of 35 subjects. Sample size was estimated using Linear Multiple Regression (F-test) with G*Power.

Data were analyzed using IBM SPSS Statistics Program (28.0v). The study team calculated the Cronbach's Alpha and McDonald’s Omega as internal consistency indexes and obtained the means and standard deviations of the measurements (see Table 2). To verify internal consistency, values ​​were supposed to be greater than 0.70 [29]. Correlation analyzes were performed between the dependent and independent variables using Pearson's Product-Moment Coefficient (*r*). To interpret the associations, the team used Taylor’s [30] classification where correlations are considered low (0.01 to 0.35), moderate (0.36 and 0.67), high (0.68 and 0.89), or very high (0.90 <). Linear multiple regression was performed. Only dependent variables (knowledge and attitudes) were included in the model as possible predictors. Sociodemographic variables were not included in the model for being nominal variables. The effect size of the predictor on the predicted variable was established through the standardized regression coefficient (β). The values ​​of β as trivial effect size (> 0.09) were classified as: small (0.10 and 0.29), medium (0.30 and 0.49), large (0.50 and 0.69), and very large (0.70 <) [31]. All results were considered significant for *p* values ​​ < 0.05. Linear regression analysis was performed considering only the LGB + sample.

## Results

### Participants

A total of 35 lesbian women, 52 gay men, 25 bisexual + women, and 8 bisexual + men completed the study. In this group, the majority (93.6%) identified their gender as cisgender. Ages ranged from 19 to 59 (*M* = 28.86, *SD* = 8.30). Almost half of the sample (48.4%) were single, and 68% percent had an annual salary of $12,000 or below, although 50.4% had a bachelor’s degree. Finally, 44.3% informed to have a religious affiliation (see Table 1).

**Table 1:** Demographic Characteristics of the Sample

**Variables**			
	**LGB + ** ***n* = 125** ***f* (%)**	**Hetero** ***n* = 75** ***f* (%)**	***p***^**1**^
Gender Identity			0.001*
Men	61 (48.8)	9 (12.0)	
Women	64 (51.2)	66 (88.0)	
Gender Expression			0.001*
Masculinity	58 (46.4)	66 (88.0)	
Femininity	59 (47.2)	8 (10.7)	
Transgender	1 (0.8)	1 (1.3)	
Non-binary	7 (5.6)	0 (0.0)	
Sexual Orientation			-
Heterosexual	-	75 (100)	
Lesbian or Gay	87 (69.6)	-	
Bisexual/Pansexual	38 (30.4)	-	
Partner/s			0.371
Yes	63 (50.8)	43 (42.6)	
No	61 (49.2)	32 (57.4)	
Individual Income			0.422
None	28 (22.4)	12 (16.0)	
< $12,000	48 (38.4)	21 (28.0)	
$12,001—$30,000	28 (22.4)	22 (29.3)	
$32,001—$50,000	12 (9.6)	11 (14.7)	
$52,001—$72,000	6 (4.8)	7 (9.3)	
$72,001—$92,000	1 (0.8)	1 (1.3)	
> $92,001	2 (1.6)	1 (1.3)	
Education Completed			0.047
Hight School	11 (8.8)	4 (5.3)	
Undergraduate degree	71 (56.8)	32 (42.7)	
Graduate degree	43 (34.4)	39 (52)	
Religious Affiliation			0.008*
No	66 (54.1)	26 (34.7)	
Yes	59 (45.9)	49 (65.3)	
Age			0.001*
19—28	82 (65.6)	27 (37.0)	
29—38	30 (24.0)	20 (27.3)	
39—48	4 (3.2)	13 (17.9)	
48—58	8 (6.4)	9 (12.4)	
59—68	1 (0.8)	4 (5.3)	

^1^*p*-values were obtained using Pearson Chi Square Test.

*Statistically significant values (*p* < 0.01)

Regarding therapy experience, 92% of the sample received services from a clinical psychologist, 8.3% from a psychiatrist, 9.9% a counseling psychologist, 4.1% a clinical social worker, and 1.7% did not know. Only 32% of the therapists disclosed their sexual orientation, 14% disclosed their religion, and 7.4% used religion and religious text as part of the therapy process. Almost three quarters of participants came out to their therapist during the initial appointment, 16.5% waited some sessions, and 9.1% never did. In addition, 43.8% of the therapists did not explore sexual orientation in the process.

The comparative sample consisted of 75 participants, with the majority being women (64%) who identified as cisgender (98%) were women Ages ranged from 19 to 63 (*M* = 34.82, *DS* = 12.38). Compared to the LGB + sample, just 28.9% were single; however, it was also the most reported option. Only 43.4% of participants had an annual salary of $12,000 or below, and the majority also had a bachelor’s degree (35.5%). Lastly, 65.8% informed to have a religious affiliation (see Table 1).

Analysis showed that all scales obtained acceptable values ​​of internal consistency higher than 0.80. As the team had a non-probabilistic sample, a Levene’s test was performed to test the homogeneity of variances. The test indicated unequal variances (*F* = 845, *p* = 0.359); therefore, a parametric test was executed (see Table 2).

**Table 2:** Cronbach’s Alpha, McDonald’s Omega Coefficient, Means, Standard Deviations, and Correlations Between the Variables

Instruments	*α*	*Ω*	*M*	*SD*	1	2	3
MHSIP-CS^1^	.94	.94	22.48	10.39	–-		
APTLGBS^2^	.96	.95	73.70	27.64	.72^**^	–-	
LGB Knowledge^3^	.89	.90	31.50	11.52	.66^**^	.95**	–-
LGB Attitudes^4^	.94	.93	44.31	17.68	.71^**^	.97**	.86**

*MHSIP-CS* = MHSIP Adult Consumer Survey, *APTLGBS* = Attitudes Perceived in Therapy toward Lesbian, Gay and Bisexual Scale, *SO* = Sexual Orientation

** = p < .01

*α* = Cronbach’s Alpha; *Ω* = McDonald’s Omega Coefficient (*n*^*1*^ = 200; *n*^*2,3,4*^ = 125)

A one-way ANOVA between groups (heterosexual vs LGB +) was conducted to compare sexual orientations in therapy satisfaction. Sexual orientation did not substantially impact therapy satisfaction (*F* (1, 198) = 0.004, *p* = 0.953). Tukey’s HSD Test for multiple comparisons found that the mean value of therapy satisfaction was not significantly different between the stratified groups: heterosexual orientation and homosexual orientation (*p* = 0.98, 95% C.I. = -3.55, 4.23) or bisexual/pansexual orientation (*p* = 0.86, 95% C.I. = -6.00, 3.83).

Results of the Pearson Correlation indicated that there was a considerable positive association between therapy satisfaction and the perception that the therapists possess adequate LGB + knowledge and positive attitudes (*r* = 0.72, *p* < 0.001). Subscales were also tested and revealed an important positive association between therapy satisfaction and the client/patient’s perception that the therapist has adequate LGB + knowledge (*r* = 0.66, *p* < 0.001) and positive attitudes (*r* = 0.71, *p* < 0.001).

A multiple linear regression analysis showed that the perception of the therapist’s LGB + knowledge and positive attitudes toward this community were significant predictor variables for therapy satisfaction. The results of the multiple regression indicated that these two predictors explained 52% of the variance (*R*^2^ = 0.519, *F*(2, 95) = 1.173, *p* < 0.001). In this model, it was found that LGB + knowledge did not significantly predict therapy satisfaction (β = 0.213, *p* < 0.116), but positive LGB + attitudes did (β = 0.329,* p* < 0.001) (see Table 3).

**Table 3:** Regression Coefficients and Associations Between Therapy Satisfaction and the Perception of Therapists’ Sexual Orientation Knowledge and Attitudes

Variables	*B*	*SE*	*β*	*t*	*p*	95% CI
Therapy Satisfaction^*α*^ [*R* = .721; *R*^*2*^ = .519; *F* = 51.281]						
Constant	1.173	2.293		.511	.610	[-3.380, 5.726]
LGB knowledge	.213	.135	.224	1.585	.116	[-.054, .481]
LGB attitudes	.329	.090	.519	3.678	.001	[.152, .507]

(*n* = 125)

## Discussion

This research has shed light on an unexposed topic in Puerto Rican sexual minorities research. Even though the attitudes of therapists and LGB + persons have been researched in the past, there has not been research regarding therapy satisfaction in Puerto Rico. International literature has shown the importance of therapists having affirmative knowledge and awareness towards LGB + clients/patients and culture [32, 33].

More than half of the LGB + sample of this study has been treated by two or more therapists, which is important data that validates previous research stating that LGB + people access mental health providers at a higher rate than heterosexual peers [34]. In both instances, therapists did not openly express their sexual orientation, yet some did express their religious beliefs. Most participants did not initially reveal their sexual orientation.

On the other hand, therapists explore if disclosing their sexual orientation might be an ethical decision that benefits the client/patient [35]. In this study, some therapists appeared to evade important, reported therapeutic recommendations for LGB + clients/patients such as asking sexual orientation and disclosing their own sexual orientation when needed. LGB + patients/clients who believed or knew that their therapist identifies as gay, lesbian, or bisexual + perceived them as being more helpful. A quote from a participant from the Kelley [36] study may exemplify this point: “Working with a gay/lesbian therapist was the smartest move. A heterosexual therapist is unable to grasp the psychic, interior, cultural dynamics of being gay and growing up in a heterosexual society.” Regarding the present study, it is possible that the small number of clients/patients who chose not to reveal their sexual orientation in therapy demonstrate the difficulty and fear that some clients/patients can experiment towards coming out, even in a therapeutic setting.

The results obtained in this study reveal that sexual orientation has no significant association with the participants’ therapy satisfaction, contradicting the first hypothesis developed by the investigators. LGB + clients/patients can go to therapy for many reasons, not all of which are inherently sexual orientation issues. For example, clients/patients may be somewhat satisfied with therapeutic interventions related to a grief process or a social skills intervention, if the therapy is limited to that event. Nevertheless, the second hypothesis about the relation between therapy satisfaction levels and the perception of knowledge and attitudes has been sustained.

The results of this study suggest that it is important for therapists to show openness, competence, and knowledge towards LGB + culture. These results also show that treating a person without considering LGB + culture is a disservice to this population. As therapists, it is important to understand the differences of the people being assisted and the intersections of those differences in a sensible and affirmative manner, especially when working with the LGB + community. While therapists approach how to treat minority populations, they must start looking at models of cultural humility [37]. Therapists need to be educated on LGB + topics and continue to learn about cultural shifts and social changes that affect this community.

This research adds to the literature review that stresses the importance of including sexual orientation education in therapist curriculums as core content [20]. Regarding cultural standards, continuous education efforts on LGB + issues should be supported to add to the formation of affirmative model professionals. Unsolicited referrals, maltreatment, and conversion efforts can have a negative effect on a person’s mental well-being [38]. Public policy on LGB + treatments should be stated in every service location. Therapists should be informed on what to expect and what to avoid regarding professional services, to prevent offering therapies that may jeopardize their patients/clients’ mental health.

The study’s strengths and limitations must be acknowledged. Some of the strengths were: 1) we have a statistically representative sample of lesbian, gay, and bisexual/pansexual individuals, 2) the study ensured a comparative group of heterosexual individuals that allows group comparisons, and 3) internal consistency of the instruments were all adequate. Study limitations must be also considered when interpreting these findings: 1) all data were cross-sectional; therefore, conclusions about causality cannot be drawn (for example, we cannot conclude that knowledge about LGB + topics causes a greater satisfaction in therapy), 2) all data were collected through self-reports and could be influenced by social desirability, minimization, or over-reporting (however, some research suggests that self-reported data of sensitive issues collected via technology devices may reduce reporting bias [39]), and 3) samples, although showing unequal variances, were significantly different by gender identity, gender expression, religious affiliation, and age; yet, studies with LGB + samples in Puerto Rico tend to have higher participation of gay men, gender diverse individuals, and report less religious affiliations [18]. Still, we recommend ensuring a higher participation of heterosexual men for future research studies, or even stratified samples.

Another limitation of the study is related to how the concept of minority stress was understood and applied. Meyer [20] highlighted that minority stress has distal and proximal components. The proximal is related to internal cognitive processes, which likely include the individual perceptions, expectations, and internalized conflicts regarding their LGB + identity. It could be possible that LGB + participants perceived negative attitudes towards them from their therapists when that was not the case. It could be suggested that some LGB + individuals, by virtue of proximal stress processes, distorted or misinterpreted their interactions with their therapists. Our study cannot answer in a definite way if there is a deficit or problem with the therapist’s knowledge or attitudes, as we did not study, asked, or observed their behavior. The data point to the fact that some LGB + participants perceived such negative attitudes.

Other research efforts should be made to continue highlighting the importance of affirmative psychological therapy services, especially being aware of our own bias, knowledge, and competences. Other variables concerning the affirmative model should be addressed in future studies, for example, examining the perception of power dynamics, discrimination, micro/meso/macro-aggressions, stereotype beliefs, and privilege awareness. Qualitative or mixed methods research should also be done to examine in-depth the barriers and positive experiences for therapy services in Puerto Rico among lesbian, gay, and bisexual/pansexual individuals. Similar research including or specifically aimed at gender minorities are also encouraged.

## Data Availability

The datasets used and/or analyzed during the current study are available from the corresponding author on reasonable request.
